# Control‐point‐specific plan robustness in volumetric modulated arc therapy‐based cranial radiotherapy

**DOI:** 10.1002/acm2.70447

**Published:** 2026-01-02

**Authors:** Daniel Crawford, Cody Church, Robert Lee MacDonald

**Affiliations:** ^1^ Department of Physics and Atmospheric Science Dalhousie University Halifax Nova Scotia Canada; ^2^ Department of Medical Physics The Ottawa Hospital Ottawa Ontario Canada; ^3^ Department of Medical Physics Nova Scotia Health, Queen Elizabeth II Health Sciences Centre Halifax Nova Scotia Canada; ^4^ Department of Radiation Oncology Dalhousie University Halifax Nova Scotia Canada

**Keywords:** control point, cranial radiotherapy, DVH, intrafraction motion, plan robustness, VMAT

## Abstract

**Background:**

Progress in mitigating plan degradation due to intrafraction patient motion may involve the identification and management of specific control points that are sensitive to motion. Robust planning in this manner could improve deliverable dosimetry and support advancements toward reducing planning target volume (PTV) margins.

**Purpose:**

To improve radiotherapy plan quality robustness in the presence of intrafraction motion by identifying the control‐point‐specific dosimetric sensitivities. This work explores control‐point‐specific plan characteristics that impact dosimetry by retrospectively assessing the consequence of simulated patient scenarios for cranial radiotherapy.

**Methods:**

Single target cranial volumetric modulated arc therapy (VMAT) treatment plans (*n* = 30) were converted into static field plans and reconstructed by applying 3D control‐point‐specific motion traces (*n* = 100) using our in‐house MATLAB application. PTV coverage (volume covered by 100% of the prescription isodose, VRx) and the differences in minimum dose delivered to 99% (D_99%_) of the gross tumor volume (GTV) were examined across the patient cohort as these are pertinent metrics for each structure. To identify the individual control points where motion led to target coverage loss, three patient plans (5 and 14 were randomly chosen, and 19 with the greatest range in prescription dose coverage) were selected for an area under the curve (AUC) analysis of control point dose volume histograms (DVHs). The mean dose difference in the area under the curve of control point DVHs (mAUC), and the standard deviation of differences (sAUC) were the metrics used in the investigation. Multileaf collimator (MLC) aperture areas were also explored as a function of these metrics.

**Results:**

Under conditions of simulated intrafraction motion, PTV coverage spanned from −2.8% to +0.73% of target volume with 78.6% of the three thousand motion traces resulting in coverage loss. There were no changes in GTV D_99%_ that exceeded ± 1.5%. For the in‐depth control point analysis, MLC aperture areas formed weak to moderately weak correlations with sAUC (*r* = −0.19, *r* = −0.42, and *r* = −0.32, *p* < 0.01 for patient plans 5, 14, and 19 respectively). In addition, two statistically distinct sub‐populations of MLC aperture areas were confirmed by Welch corrected *t*‐tests (*p* < 0.0001, *p* = 0.02, *p* = 0.005 for cases 5, 14 and 19) across a threshold of ± 0.05 mGy in mAUC.

**Conclusion:**

This work has demonstrated that the dosimetric impact of intrafractional motion reflects the inherent motion sensitivity of specific control points. Our findings suggest that motion sensitive control points could be selectively targeted for gating to enhance robustness against intrafraction motion and improve dosimetry in support of a PTV margin reduction strategy. Single target cranial plans serve as ideal cases to characterize the consequences of motion at the control point level with the aim of expanding the analysis to other anatomical regions.

## 1 INTRODUCTION

The dynamic processes of volumetric modulated arc therapy (VMAT) promote precise and accurate dose delivery by producing a conformal dose distribution to the target with steep dose gradients for organs at risk (OAR) sparing. VMAT plans are created via control points located at discrete geometric locations, each defining parameters such as the MLC leaf positions, jaw positions, monitor units, and collimator angle which together form the treatment geometry along the arc. A well‐known complication and source of dosimetry error is intrafraction motion which adds uncertainty in delivered dose, resulting in blurring of the planned dose distribution.^1^

Modern frameless cranial radiotherapy utilizes thermoplastic masks to immobilize the patient, which are custom molded to minimize patient motion and accurately reproduce patient positioning during fractionation. While this immobilization strategy is effective and widely used, motion is not eliminated. Investigations have shown that variations in positioning accuracy can range from 0.5 to 4 mm, and displacements within frameless systems exceeded 3, 2, and 1 mm in 1%, 3%, and 12% of 967 treatment sessions respectively.^2^, ^3^ Intrafraction motion is common, poses a nonnegligible impact on dose delivery, and is known to increase with treatment duration.^4^, ^5^ As intrafraction motion can produce a discrepancy between planned and delivered dose distributions, it introduces uncertainty in target coverage. To mitigate both inter‐fraction and intra‐fraction uncertainty, margins are added to the target volume.^6^ Within stereotactic radiotherapy (SRT) and stereotactic radiosurgery (SRS), PTV margins typically range between 0 and 3 mm depending on delivery system, motion management technique, and clinical protocols.^7^, ^8^, ^9^, ^10^

Dose volume histograms (DVHs) are used throughout the treatment planning process to evaluate the dose delivered to contoured volumes. Researchers compared the DVHs from five treatment planning systems (TPS) and concluded the median precision among the systems ranged from 0.902% to 3.22%.^11^ While the variability may have implications for plan evaluation and plan comparison, it may also be a concern for robustness assessments that rely on independent DVH calculations performed outside of a TPS. Conventional treatment plans are typically formulated and evaluated under the assumption of perfect patient positioning and stationary anatomy. Patient motion and variability are generally managed by the addition of planning margins which include surrounding healthy tissue. Robustness efforts characterizing the consequence of patient motion through the simulation of patient scenarios often describe DVH bands, where ranges of DVHs are plotted around a static or nominal DVH, with worst‐case scenarios revealing the extrema of plan vulnerability to motion, highlighting a risk for target underdosing or OAR overdosing.^12^, ^13^

The aim of this work is to explore the consequence of realistic 3D simulated intrafraction motion on cumulative and control‐point‐specific dose distributions of single target cranial radiotherapy cases for potential PTV margin reduction. We compiled work from our prior research to: (1) apply realistic motion traces derived from a previously acquired patient motion tracking investigation^14^ and (2) examine PTV coverage of cumulative dose distributions with three patient plans selected for an AUC analysis of control point DVHs. Researchers have developed and applied AUC methods however, their work centered on plan‐level cumulative dose distributions rather than control‐point‐specific analyses.^15^, ^16^, ^17^ We considered mAUC and sAUC to identify motion sensitivity at individual control points. While no single patient motion trace is expected to be accurate, the population statistics of the deviations at control points are representative of large population trends. These statistics should be indicative of plan robustness, as it relates to non‐uniform coverage loss with potential consequences for PTV margin reduction. Utilizing these metrics (mAUC and sAUC) to understand plan robustness as it pertains to GTV coverage is important to support margin reductions. However, the relationship between mAUC, sAUC and GTV coverage have yet to be explored in the literature.

## 2 MATERIALS AND METHODS

### 2.1 Static field plan reconstruction

Thirty previously treated single target cranial VMAT plans were exported from Eclipse v18.0 and deconstructed to into a plan composed of static fields for each control point using an in‐house MATLAB v 2023a (MathWorks, Natick, Massachusetts, USA) application.^18^ All plan parameters (prescription dose (Rx), monitor units, MLC configuration etc.) remained the same as the original clinical plan. The dose grid was set to 1.5 mm with Anisotropic Analytical Algorithm (AAA) v15.6.05 selected to calculate dose volumes to reduce computational burden in the anatomical region where tissue density is approximately homogeneous.^19^, ^20^, ^21^ All plans were previously optimized using Eclipse, with treatment delivered on Varian TrueBeam linear accelerator equipped with a high‐definition 120‐leaf multileaf collimator (HD120 MLC). The patient group (Table 1) included one stereotactic radiosurgery (SRS) case consisting of a single 20 Gy fraction and 488 control points; twelve stereotactic radiotherapy (SRT) cases with doses per fraction ranging from 5 to 10 Gy with the number of control points ranging from 294 to 488; and seventeen conventionally fractionated cases with doses per fraction between 1.8 and 2.5 Gy and number of control points ranging from 292 to 488.

**TABLE 1:** Plan characteristics for the thirty‐patient cohort.

Plan characteristics across 30 patients				
PTV vol (cc)	Freq.	Rx (Gy)	VMAT arcs	Control points
0–5	9	20–50	3–4	310–488
5–10	6	20–50	2–4	356–488
10–20	6	25–50	2–4	292–488
20–30	2	50	3–4	374–488
30–40	2	30–50	3–4	374‐488
40–50	1	27	3	294
50–60	1	54	4	472
60–70	3	30–56	3–4	326–488

Abbreviation: Freq. Frequency.

### 2.2 Simulated intrafraction motion traces

**FIGURE 1:**
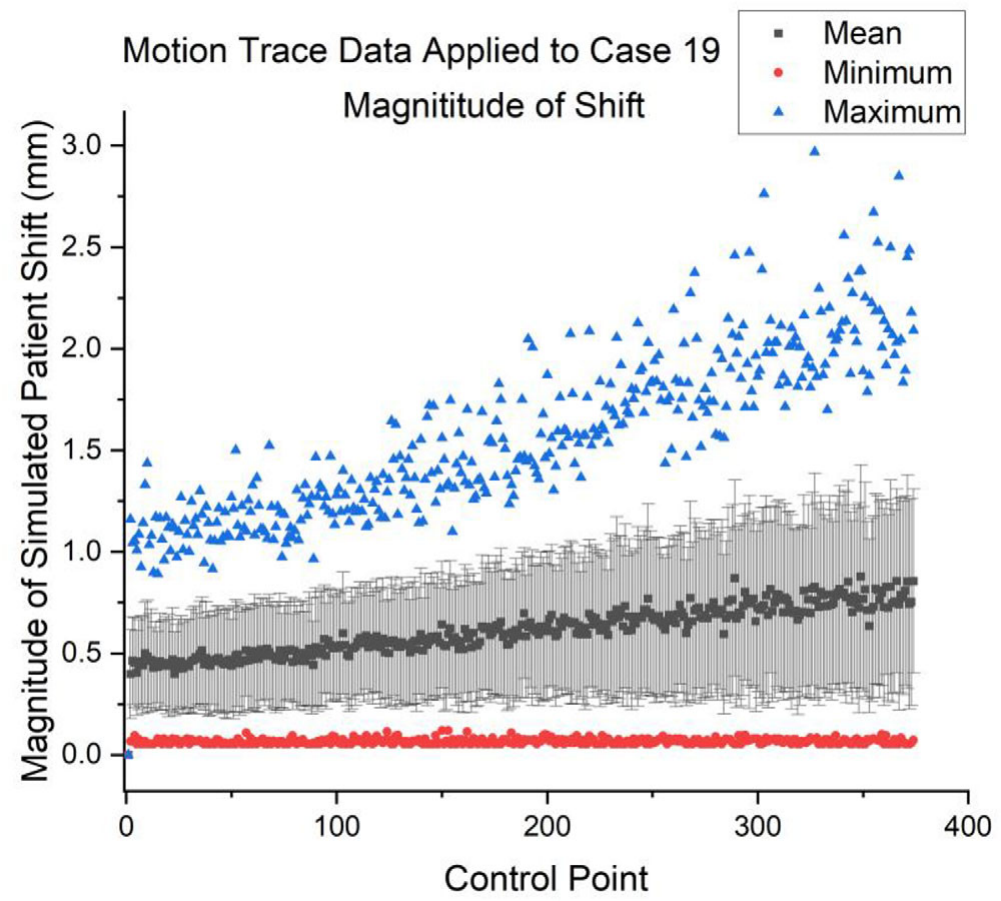
Simulated motion trace data applied to patient case 19 with the magnitude of dose volume displacement not exceeding 3 mm. In our modeling, the magnitude of motion increased with treatment duration.

To assess the consequences of intrafraction motion on single target cranial patients immobilized in thermoplastic masks, previously published 3D motion tracking probability distribution functions were used to generate one hundred control‐point‐specific time evolved 3D motion traces (Figure 1).^14^ Consistent with intrafraction motion increasing with treatment duration, a treatment duration estimate using a gantry speed of 6 degrees per second was aligned with the previously published data to modulate the range of simulated patient motion (Table 2). These traces were input as shifts to reconstruct the static‐field plan simulating one hundred patient motion scenarios. Rotational motion was not modeled as rotational data was not included in the population data referenced to generate the motion traces. However, in the geometry of single target cranial cases where the isocenter is located at the GTV centroid, we believe small rotations can be reasonably approximated by 3D rigid translation.

**TABLE 2:** Parameters to generate realistic 3D motion traces simulating patient scenarios.

Direction	Means	Standard deviations	Initial condition	
*X*	−0.072	1.08	Limit	<0.05 mm
*Y*	−0.207	1.06		
*Z*	0.195	1.58		

Applying motion traces to control points approached an intensive feasibility limit within this computational method, making it desirable to identify any potential surrogates for control point sensitivity that may reduce computational burden.

### 2.3 DVH analyses

The method in this work for DVH analysis was external to the treatment planning system and was previously validated by Monte Carlo simulation.^22^, ^23^ Cumulative DVHs were produced by the summation of dose matrices over all control points with a fivefold increase in dose grid resolution using linear interpolation, as this super sampling provided the best agreement with DVHs in the TPS.

The distribution of prescription dose coverage for PTVs with 2 mm margins was examined, and the GTV D_99%_ was assessed ([transformed—static]/static) * 100) for all motion traces across all patients. Case #19, associated with the greatest deviation in V_Rx_, along with two randomly selected patient cases (5 and 14), were chosen for further examination at the control point level in attempt to quantify motion sensitivity of control points (Table 3).

**TABLE 3:** Plan characteristics for three cases selected for control‐point analysis.

Plan Characteristics for cases 5, 14, and 19					
Case	PTV vol (cc)	Rx (Gy)	Fractions	VMAT arcs	Control points
5	6.84	20	8	3	374
14	14.12	50	25	2	292
22	5.24	25	5	3	390

To identify which control points within the treatment plans contributed to PTV coverage deterioration, the AUC of control point DVHs of the PTV were examined. A mean dose difference threshold of ±0.05 mGy per control point, corresponding to approximately one standard deviation in the population of mAUC, was imposed to separate motion sensitive and motion resistant subcategories of control points. With respect to the standard deviation of dose differences, MLC aperture areas within each subcategory were examined as a function of sAUC in effort to further explore potential trends in control point sensitivity to motion. A Welch corrected *t*‐test was conducted to assess the difference between subpopulations of MLC aperture areas. In all, a total of three thousand cumulative DVHs were analyzed across all thirty patients, and 105,100 control point DVHs for the three patient cases were analyzed in OriginLab (Version 2024b, OriginLab Corporation, Northampton, Massachusetts, USA).

## 3 RESULTS

**FIGURE 2:**
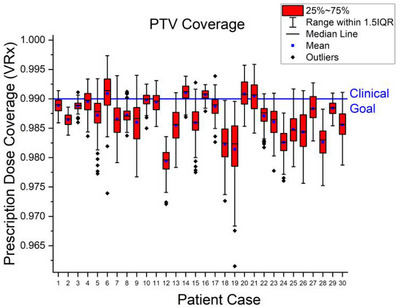
Simulated intrafraction motion resulted in deviations in PTV coverage ranging from +0.7% to −2.8% of target volume with respect to the V_Rx_ = 99% clinical coverage goal. The 2 mm PTV expansion of the clinical target volume remains sufficient to accommodate coverage of the GTV in the presence of patient motion.

Simulated motion resulted in observable banding when cumulative DVHs were plotted alongside the static cumulative DVH. Banding is a consequence of simulated motion‐induced variability in dose deposition. As motion traces were applied to control point dose volumes, the dose received by each voxel had the potential to vary slightly across patient scenarios, resulting in subtle fluctuations in the cumulative DVH. Across the patient cohort, the populations of V_Rx_ approximated normal distributions with 2359 motion traces out of 3000 (78.6%) resulting in some amount of coverage loss. The greatest observed deviation from the V_Rx_ clinical goal (99% of PTV covered by prescription) was a 2.8% reduction in coverage associated with case 19 (Figure 2).

**FIGURE 3:**
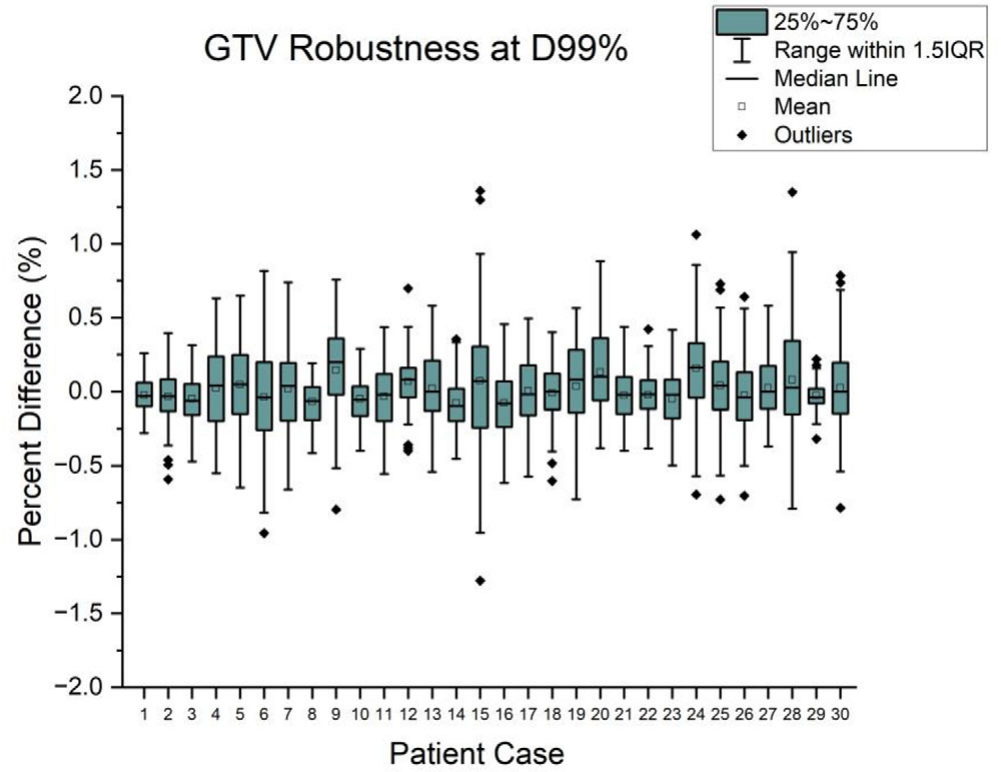
Variability in GTV D_99%_ was assessed for all patient cases. The smallest deviation occurred with case 29, spanning 0.2% to −0.3%. The greatest observed differences were associated with patient case 15, ranging from +1.4% to −1.3%.

The prescription normalized differences in GTV coverage (D_99%_) were within ± 1.5% relative to the static clinical plans. The largest differences were observed in patient case 15 (PTV = 4.32 cc, Rx = 50 Gy) which spanned −1.3% to 1.4% (Figure 3). On inspection, a small section of the GTV overlapped with the brainstem contour, suggesting a possible directional sensitivity.

**FIGURE 4:**
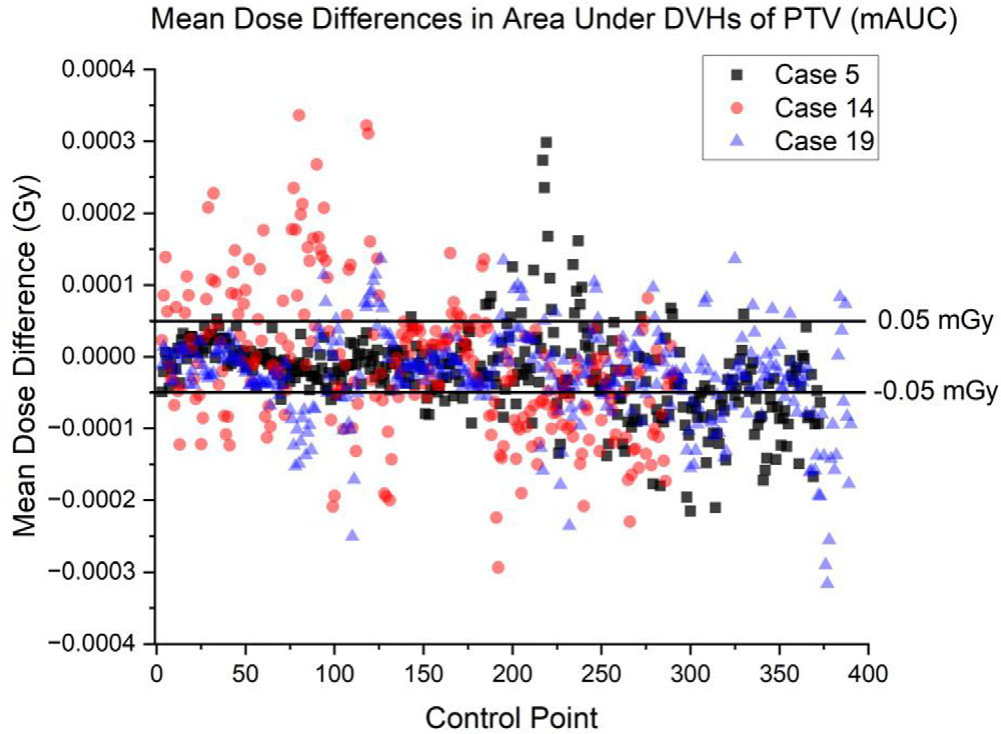
For case 5 (black), 14 (red), and 19 (blue), the mAUC shows the magnitude of differences from the clinical plan with variability between control points. Control points were partitioned across a threshold of ± 0.05 mGy in mAUC to isolate motion sensitive control points and explore the possibility of subpopulations of MLC aperture areas.

Cases 5, 14 and 19 were selected for an analysis of DVHs at individual control points. To examine the presence of subpopulations of MLC aperture areas, a threshold at ± 0.05 mGy in mAUC partitioned the relatively motion sensitive from motion resistant control points (Figure 4).

**FIGURE 5:**
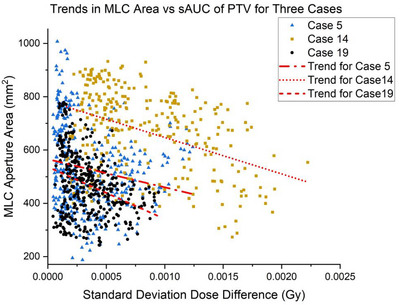
For cases 5, 14 and 19, MLC aperture areas are presented as a function of sAUC of control point DVHs. The combined case data has shown weak to moderate correlations of *r* = −0.19, *r* = −0.42, and *r* = −0.32 for cases 5, 14, and 19, respectively.

An examination of MLC aperture areas as a function of sAUC (Figure 5) formed weak to moderately weak correlations for cases 5 (*r* = −0.19; 95% CI: –0.28 to –0.09), 14 (*r* = –0.42; 95% CI: –0.51 to –0.32), and 19 (*r* = −0.32; 95% CI: –0.41 to –0.23). However, the combined case data formed a significant (*p* < 0.01) relationship with sAUC of the 105,100 control point DVHs.

**FIGURE 6:**
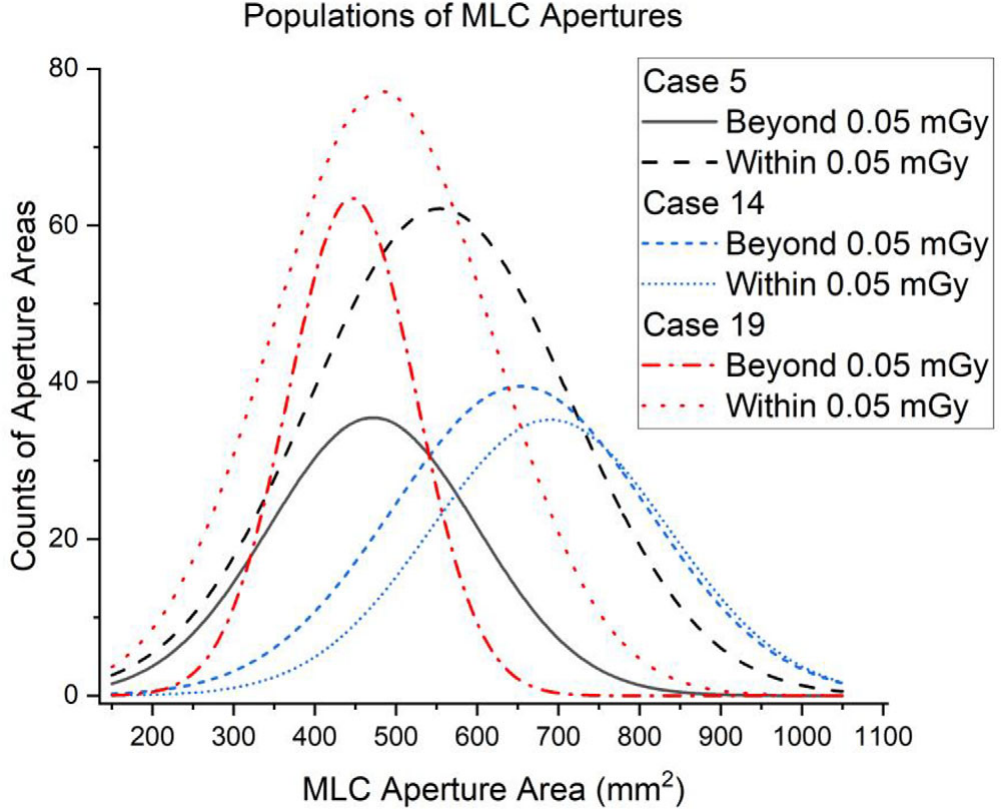
Welch corrected t‐tests revealed the presence of two statistically different sub‐populations of MLC aperture areas partitioned by ±0.05 mGy in mAUC of control point DVHs (*p* < 0.0001, *p* = 0.02, *p* = 0.005 for case 5, 14 and 19, respectively). The assumption of equal variance produced a similar result.

Welch's *t*‐tests confirmed the presence of two statistically different subpopulations of MLC aperture areas within the treatment cases (*p* < 0.0001, *p* = 0.02, *p* = 0.005 for plans 5, 14 and 19 respectively) (Figure 6).

Our in‐house MATLAB application was developed for central processing unit (CPU) computation on a standard workstation without algorithmic optimization. The per patient processing time was on the order of twenty‐four hours with output data exceeding 80 GB.

The maximum speed of Varian TrueBeam Linacs is 6°/s which was used as an estimate knowing the gantry slows for modulation in stereotactic radiosurgery (SRS).^24^ There was only one SRS and one hypofractionated plan within the 30‐patient cohort.

## 4 DISCUSSION

At the plan‐level, the 2 mm PTV margins required by our protocol were sufficient to accommodate the 3D displacements simulating patient motion to ensure coverage of the GTV (Figure 3). Control point dose volume displacements did not exceed 3.5 mm within any of the three thousand applied motion traces (Figure 1). It is unlikely that a large transient shift occurring at a single control point would be solely responsible for plan level coverage degradation as the prescription is shared among hundreds of control points. However, A collection of large shifts occurring at motion sensitive control points may contribute to coverage loss.

It was found that control‐point‐specific MLC apertures may share a relationship with the inherent sensitivity of the control point. Other possible sources of sensitivity are likely a combination of beam geometry and proximal structure geometry (location and distance of OARs) in relationship with dose gradients, which may be best addressed from a machine learning perspective. Inspection of MLC apertures suggested control points with relatively small areas could serve as a computationally inexpensive surrogate for identifying control point sensitivity.

Although the reported correlations between MLC aperture areas and the standard deviation of dose differences were weak, the association is viewed as meaningful for several reasons. The selection of ± 0.05 mGy in mAUC as the threshold reasonably delineated the dataset by approximately one standard deviation, effectively separating sizeable fractions of control points by their degree of sensitivity. The current threshold is not an optimized value whereas optimization would likely stratify the MLCs and control points according to coverage loss. The relationship between plan complexity and robustness remains an important consideration, as measurable increases in correlation may translate into measurable improvements in plan robustness.^25^, ^26^ Lastly, Welch corrected t‐tests indeed confirmed the presence of two statistically different subpopulations of MLC aperture areas within each of three analyzed cases. It would be appropriate to examine control‐point MLC apertures as clusters.

Modern motion management systems have the capability of applying beam holds when a user‐defined motion deviation is reached. The AlignRT system for surface‐guided radiotherapy (SGRT) provides motion tracking in six degrees of freedom relative to a static image. Beam holds can be automated if a preset threshold is exceeded.^27^ Similarly, Varian's Identify system for SGRT offers customizable automated beam hold settings in support of personalized treatment.^28^ The presence of motion sensitive control points suggest it may be possible to apply motion tolerances at motion sensitive control points to prevent unnecessary beam holding during treatment, should this demand arise for plan quality. Together, the combination of control‐point plan robustness and motion management may form a margin reduction strategy. In recognizing an ongoing interest for PTV margin reduction, we assessed prescription coverage loss to the PTV as a means of approximating the steps necessary to manage control‐point‐specific motion in cranial radiotherapy planning scenarios, with potential implications for future gross tumor volume (GTV)‐focused approaches.

Radiotherapy plans are uniquely tailored to individual patient anatomy and clinical objectives which contribute to inter‐patient variability. In this work, we observed non‐uniform differences in the distributions of DVHs at the plan level and within individual control points revealing beam‐aperture susceptibility to simulated patient motion. The presence of motion sensitive control points within the plans can have important implications for robustness efforts, motion management and potential margin reduction strategies. Identifying highly sensitive sub‐arc segments may enable targeted plan optimization to minimize the spectrum of motion sensitivity at a granular level and reinforce plan level dose volumes. Our approach offers a dosimetrically informed strategy that can directly incorporate motion susceptibility into plan optimization. Employing tools such as the Eclipse aperture shape controller to increase MLC apertures or minimizing monitor units, represent indirect strategies to enhance robustness and potentially reduce motion sensitivity.^29^ We view our strategy as complimentary to existing methods with potential clinical relevance supported by connections between plan complexity and plan robustness.^30^, ^31^ Finally, we believe a multivariate analysis incorporating beam parameters, geometry, plan metrics, and OAR data are required to fully understand why some control points display more motion sensitivity than others.^32^

Several assumptions and limitations underlie our investigation into control point robustness. Dose volumes were rigidly translated without recalculation. A prior investigation applied intrafraction motion traces using predefined trajectories with dose volumes calculated by Monte Carlo simulation to fully capture the consequence of motion without volume recalculation.^22^ The authors reported V_100_ differences below 8 × 10^−^⁴ cc when using spatially invariant spherical phantoms. Furthermore, the cranium is a unique part of the anatomy where tissue density is relatively homogeneous, internal motion is minimal, and the tumor volume is rigid. We assume the population of rigid 3D transformations can approximate rotational motion given that the GTV center of mass lies at the isocenter for our single target cranial cases. In addition to a growth constraint applied during motion trace generation, applying a maximum gantry speed (6°/s) further limited the displacement in the simulated motion. At our clinic, control points are evenly spaced at 2° of arc to ensure the static field converted patient plans were a high‐resolution, granular representation of VMAT delivery. Our next steps focus on the identification of a surrogate indicator for control‐point sensitivity using machine learning methods and formulating a PTV margin reduction strategy that can be extended to other anatomical sites. Our findings serve as an example of an analysis for what is expected for coverage loss if the PTV margin is reduced or eliminated with the understanding that many clinics rely on GTV‐only margins.^33^ In this work, plans were generated with the prescription isodose conformal to the PTV while GTV coverage analysis requires replanning with GTV conformal prescription dose, or reduced margins.^34^

## 5 CONCLUSION

This work demonstrates that control point sensitivity to intrafraction motion has significant variation. Therefore, individual control point analyses could be a source for improving delivered dosimetry and enhancing robustness against intrafraction motion, in support of the potential for PTV margin reduction. Plan processing within the current version of our MATLAB application required considerable computational time. Clinical implementation will require surrogate indicators to identify control point sensitivity to motion which is a subject for our future work. Single target cranial plans serve as ideal cases to characterize the consequences of motion at the control point level leading to the inclusion of other anatomical regions with greater complexity.

## AUTHOR CONTRIBUTIONS

Daniel Crawford: Methodology, data collection and analysis, writing manuscript draft. Cody Church: Conceptualization, provided code, review and editing manuscript. Robert Lee MacDonald: Supervision, validation, review and editing manuscript.

## CONFLICT OF INTEREST STATEMENT

The authors declare no conflicts of interest
